# Predominant frequency of HLA‐B*27 in patients with ankylosing spondylitis in southeastern China

**DOI:** 10.1002/iid3.524

**Published:** 2021-09-09

**Authors:** JiaoJiao Lu, Jing Yang, WenXu Dong, BaoJia Tang, LuoYuan Cao, YingHua Lin, BaoYing Huang, XianGuo Fu

**Affiliations:** ^1^ Department of Central Laboratory Ningde Municipal Hospital Affiliated to Ningde Normal University Ningde Fujian China; ^2^ Department of Clinical Laboratory Ningde Municipal Hospital Affiliated to Ningde Normal University Ningde Fujian China; ^3^ Department of Traditional Chinese Medicine Ningde Municipal Hospital Affiliated to Ningde Normal University Ningde Fujian China

**Keywords:** ankylosing spondylitis, high‐resolution PCR‐SSP, HLA‐B*27, subtypes

## Abstract

**Objectives:**

This study was to investigate the polymorphism and distribution of alleles of HLA‐B*27 in patients with ankylosing spondylitis (AS) in Han population of southeastern China.

**Methods:**

A total of 89 peripheral blood samples from southeastern Chinese Han patients with AS that diagnosed according to Modified New York criteria were subtyped using the high‐resolution PCR‐SSP.Exon 2‐3 of HLA‐B*27 gene was amplified and sequenced to further confirm the HLA‐B*27 subtype.

**Results:**

The frequency of HLA‐B*27 was 99.87% in AS patients. Three subtypes, HLA‐B*2704, HLA‐B*2705, and HLA‐B*2706 were identified. The frequencies for these three alleles were HLA‐B*2704 in 84/88 (95.46%), HLA‐B*2705 in 3/88(3.41%), and HLA‐B*2706 in 1/88 (1.13%) of the HLA‐B*27 positive patients, respectively.

**Conclusions:**

Our study shows that HLA‐B*2704 has an overwhelming frequency in southeastern Chinese Han AS patients. A combined analysis including previous studies of HLA‐B*27‐subtype distributions in Chinese Han populations showed that HLA‐B*2704 may originate from the southern Han and then migrate and spread to the northern areas, and HLA‐B*2705 show the opposite result.

## 1 INTRODUCTION

Ankylosing spondylitis (AS) is an inflammatory systemic disease that primarily affects the sacroiliac joints and spine. In addition, specific organ involvement including anterior uveitis, psoriasis, and chronic inflammatory bowel disease, may simultaneously occur in AS.^1^ AS prevalence varies significantly in different parts of the world.^2^ The prevalence of AS is 0.24% in the Chinese population, similar to the incidence in populations of European ancestry, then that is roughly 0.5% in the United States.^3^ Genetic factors play an important role in the pathogenesis of AS, which is estimated to contribute up to 97% of AS susceptibility.^4^ Human leukocyte antigen (HLA)‐B*27 is strongly associated with AS and it remains amongst the strongest genetic association with any common human disease.^5^, ^6^

By now, 238 subtypes of HLA‐B*27 have been reported (The IPD‐IMGT/HLA Database, Release 3.43.0, 2021). AS has been reported to occur with the following subtypes: HLA‐B*2702, HLA‐B*2703, HLA‐B*2704, HLA‐B*2705, HLA‐B*2706, HLA‐B*2707, HLA‐B*2708, HLA‐B*2710, HLA‐B*2714, HLA‐B*2715, and HLA‐B*2719.^7^, ^8^ HLA‐B*2705, HLA‐B*2702, and HLA‐B*2704 are the most common subtypes that have been shown strong association with AS in different populations. The distribution of HLA‐B*27 subtypes varies depending on ethnicity and geographic distributions.^9^ HLA‐B*2705 and HLA‐B*2702 are the predominant subtypes in Caucasians and show a hierarchy in terms of allelic frequencies, HLA‐B*2702 shows a higher frequency in the Jewish population and North Africa Caucasians and is also found in Asians, and HLA‐B*2704 the predominant subtype in Asian populations.^7^, ^10^ It has been suggested that the differences found in the distribution of HLA‐B*27 alleles are caused by different migration streams. HLA‐B*2705 has a decreasing North‐Southeast gradient and HLA‐B*2704, which is virtually restricted to Asian populations, displays a decreasing East‐Southwest gradient.^11^, ^12^, ^13^, ^14^ In Asian populations, HLA‐B*2704 could have arisen in the Chinese population and later spread through East and South Asia.^14^ To obtain the regional differences in HLA‐B*27 alleles in Chinese population will help to understand the role for regional characteristics in the evolution of HLA‐B*27 subtypes with AS. The purpose of this study was to investigate the frequency of HLA‐B*27 subtypes with AS in the southeastern Chinese Han population and confirm its geographic distribution of HLA‐B*27 subtypes with AS in the Chinese population.

## 2 MATERIALS AND METHODS

### 2.1 Subjects

A total of 89 patients with AS (64 males and 25 females) from Han population in southern‐east China were included in this study. AS patients were diagnosed according to Modified New York criteria,^15^ written informed consent was obtained from all patients.

### 2.2 Genomic DNA isolation

Genomic DNA was isolated from the peripheral blood of the patients with QIAamp DNA blood kit (Qiagen) and quantified on BioPhotometer (Eppendorf).

### 2.3 Subtyping with PCR sequence‐specific primers (PCR‐SSP)

HLA‐B*27 subtyping was performed using the HLA‐B*27 subtyping kit (SYBR Green I RealTime polymerase chain reaction [PCR] method) (Super Biotechnology). The reaction set‐up and PCR program was according to the manufacturer's recommendations and performed on the ABI StepOne plus PCR system (Applied Biosystems). The results were analyzed using the StepOne™ software, version 2.2.2.

### 2.4 Exon 2‐3 amplification and sequencing

To further confirm the HLA‐B*27 subtype, Exon 2‐3 was amplified and sequenced using primers previously described by Voorter et al.^16^ The localization and sequence of amplification and sequencing primers are indicated in Table 1. The 50 µl PCR system consisted of 100 ng template DNA, 20 pmol of each primer, and 2.5 U AmpliTaq Gold DNA Polymerase (Applied Biosystems). The reaction was performed on an ABI 2720 PCR system using the following parameters: initial denaturation 96°C for 2 min, 30 cycles of 94°C for 30 s, 63°C for 1 min, 72°C for 1 min, and final extension step at 72°C for 10 min. All PCR products were purified with a QIAquick Gel Extraction Kit (Qiagen) and sent to Shanghai‐based companies to perform sequencing using ABI 3730XL DNA sequencer (Applied Biosystems).

**Table 1:** Amplification and sequencing primers for sequence‐based typing of exons 2 and 3

Location	Name	Sequence (5ʹ→3ʹ)	Length (bp)
Exon2	E2F	GGGAGGAGCGAGGGGACCGCAG	530
	E2R	ACTGAAAATGAAACCGGGTAAAC	
	Sequencing primer	GCTCCCACTCCATGA	
Exon3	E3F E3R	TACCCGGTTTCATTTTCAGTTG GGAGGCCATCCCCGGCGACCTAT	432
	Sequencing primer	GGGCTGACCGCGGGG	

## 3 RESULTS

In these patients, the ratio of female to male AS patients to be 1:2.56, confirming that AS is definitely a disease affecting predominantly males. In 89 AS patients, there were 88 HLA‐B*27 positive cases and 1 HLA‐B*27 negative. The frequency of HLA‐B*27 was 99.87% in AS patients.

In 88 HLA‐B*27 cases, three subtypes were identified. HLA‐B*2704, the predominant subtype, was detected in 84/88 (95.46%), HLA‐B*2705 in 3/88(3.41%), and HLA‐B*2706 in 1/88 (1.13%) of the HLA‐B*27 positive patients. The results showed that HLA‐B*2704 was the most strongly associated subtype with AS in this region of China. The results were shown in Table 2.

**Table 2:** HLA‐B*27 alleles in AS patients in Chinese Han population of different areas

Areas (number of patients)	References	HLA‐B*27 alleles *n* (%)									
		B*2702	B*2703	B*2704	B*2705	B*2706	B*2707	B*2710	B*2713	B*2715	B*27*24
Beijing (493)	Zhang et al.^17^	12 (2.4)	0	275 (55.8)	205 (41.6)	0	1 (0.2)	0	0	0	0
Shanxi (380)	Wang et al.^18^	0	0	217 (57.1)	143 (37.6)	0	11 (2.9)	0	0	0	0
Chongqing (124)	Huang et al.^19^	2 (1.59)	1 (0.79)	72 (57.14)	41 (32.53)	0	8 (6.34)	0	0	0	0
Xinjiang (152)	Zhou et al.^20^	1 (0.66)	0	93 (61.18)	48 (31.58)	0	3 (1.97)	0	0	3 (1.97)	1 (0.66)
Wuhan (172)	Liu et al.^21^	1 (0.6)	1 (0.6)	119 (69.2)	41 (23.8)	0	0	0	1 (0.6)	0	0
Suzhou (158)	Yu et al.^22^	1 (0.63)	0	116 (73.41)	38 (24.05)	0	3 (1.89)	0	0	0	0
Shanghai (130)	Liu et al.^23^	0	0	105 (80.8)	24 (18.5)	0	0	1 (0.8)	0	0	0
Guangdong (453)	Mou et al.^24^	3 (0.7)	0	395 (87.5)	49 (10.8)	0	0	0	0	6 (1.3)	0
Hunan (111)	Ma et al.^13^	0	0	98 (88.3)	12 (10.8)	0	0	0	0	0	1 (0.9)
Southern Fujian (103)	Fan et al.^25^	0	0	98 (95.1)	3 (2.9)	0	1 (1.0)	0	0	1 (1.0)	0
Current study (88)	Lu et al.	0	0	84 (95.46)	3 (3.41)	1 (1.13)	0	0	0	0	0
Taiwan (82)	Chou et al.^26^	0	0	77 (94)	5 (6)	0	0	0	0	0	0
Taiwan (416)	Hou et al.^27^	0	0	309 (98.4)	4 (1.3)	1 (0.3)	0	0	0	0	0
Hainan (41)	Xu et al.^28^	0	0	41 (100)	0	0	0	0	0	0	0

Abbreviation: AS, ankylosing spondylitis.

## 4 DISCUSSION

AS has a high disability rate and insidious morbidity, therefore early diagnosis of AS is of great significance. The New York criteria revised in 1984 is still used for the diagnosis of AS.^29^ The diagnosis of AS mainly depends on imaging and clinical manifestations. When patients present with typical imaging changes and clinical manifestations, it is often advanced to the middle and late stages, leading to delayed diagnosis and treatment.^30^ Studies have found that AS is highly correlated with HLA‐B*27, which can be used as one of the important indicators for early diagnosis of AS. Across the world, the distribution of AS varies by ethnic and region. Therefore, to diagnose AS at an early stage, we should consider the distribution characteristics and rules of subtypes in this area, and carry out the corresponding subtype detection.

In current study, we detected an overwhelming frequency of HLA‐B*2704 in AS patients in the Han population in the southeastern Fujian region, accounting for 95.46%. This is consistent with other findings in this province and Taiwan region.^25^, ^26^, ^27^ The other two subtypes are HLA‐B*2705 and HLA‐B*2706 with frequencies of 3.41% and 1.13%, respectively. By summarizing the recent research data from several regions in China, the results showed that there were differences in the distribution of HLA‐B*27 gene subtypes in AS patients from different regions, HLA‐B*2704 and HLA‐B*2705 are the predominant subtypes of AS patients. The data also showed that the HLA‐B*27 subtypes of AS patients in China presented a regular distribution from the geographical space.

It has been suggested that the differences found in the distribution of HLA‐B*27 alleles are caused by different migration streams.^14^ In Asian populations, HLA‐B*2705 has a decreasing North‐Southeast gradient.^11^, ^12^, ^13^, ^14^ It may be due to North Euro‐Asian migrations introducing this subtype into southern regions.^14^ HLA‐B*2704 is the predominant subtype in the Asian population and displays a decreasing East‐Southwest gradient, indicates that this subtype could have arisen in the Chinese population and later spread through East and South Asia.^14^ Migration has a linear effect on gene frequencies and Neolithic demic diffusion could be the possible cause of the many continent‐wide genetic gradients.^14^ The data of this study combined with the data of previous studies (in Table 2) could inferred that HLA‐B*2704 may originate from the southern Han and then migrate and spread to the northern areas, and HLA‐B*2705 show the opposite result.

All HLA‐B*27 subtypes differ by only a few amino acid, HLA‐B*2706 differs from HLA‐B*2704 by amino acid changes at only two residues, 114 and 116 of the heavy chain.^31^ In accordance with the WHO Nomenclature Committee for the HLA System, this allele should be named HLA‐B*2722.^32^ HLA‐B*2706 (HLA‐B*2722) is a rare subtype identified only in some Southeast Asian populations.^33^ It has been described that HLA‐B*2706 (HLA‐B*2722) was negatively correlated with AS, and generally considered to be the protective subtype.^34^ In our study, one AS patient was identified as the HLA‐B*2706 subtype. The subtype was also reported in AS patients from the Chinese population.^10^, ^27^ Therefore, we cannot determine whether HLA‐B*2706 is negatively associated with AS in the Chinese population. In our study, one AS patient's HLA‐B*27 subtyping was negative, which suggests that other pathogenic agents may also be involved in the pathogenesis of AS, such as aminopeptidase gene, IL‐23R, Type 17 immunity, intergenic regions and gut microbiom.^35^, ^36^, ^37^

## 5 CONCLUSION

In conclusion, HLA‐B*2704 was the predominant and most strongly associated subtype with AS in Han population in Southeastern China, which increases gradually from north to south, while HLA‐B*2705 showed a decreasing trend from north to south. HLA‐B*2706 might be not the protective subtype in the Chinese population, which needs to be investigated with a larger group of patients with AS and controls.

## AUTHOR CONTRIBUTIONS

JiaoJiao Lu collected the data and wrote the article. Jing Yang, WenXu Dong, and BaoJia Tang carried out the experiment. LuoYuan Cao arranged and analyzed experimental data. YingHua Lin and BaoYing Huang were in charge of article modification and XianGuo Fu was in charge of project guidance and experimental analysis. All authors read and approved the final manuscript.

## CONFLICT OF INTERESTS

The authors declare that there are no conflict of interests.

## Data Availability

The data that support the findings of this study are available from the corresponding author upon reasonable request.

## References

[1] Chen B , Li J , He C , et al. Role of HLA‐B27 in the pathogenesis of ankylosing spondylitis (review). Mol Med Rep. 2017;15(4):1943‐1951. 28259985 10.3892/mmr.2017.6248 PMC5364987

[2] Pedersen SJ , Maksymowych WP . The pathogenesis of ankylosing spondylitis: an update. Curr Rheumatol Rep. 2019;21(10):58. 31712904 10.1007/s11926-019-0856-3

[3] Ng SC , Liao Z , Yu D , et al. Epidemiology of spondyloarthritis in the People's Republic of China: review of the literature and commentary. Semin Arthritis Rheum. 2008;37(1):39‐47. 10.1016/j.semarthrit.2007.01.003 17350674

[4] Braun J , Bollow M , Remlinger G , et al. Prevalence of spondylarthropathies in HLA‐B27 positive and negative blood donors. Arthritis Rheum. 1998;41(1):58‐67. 9433870 10.1002/1529-0131(199801)41:1<58::AID-ART8>3.0.CO;2-G

[5] Brown MA . Breakthroughs in genetic studies of ankylosing spondylitis. Rheumatology. 2008;47(2):132‐137. 18037607 10.1093/rheumatology/kem269

[6] Robinson PC , Brown MA . Genetics of ankylosing spondylitis. Mol Immunol. 2014;57(1):2‐11. 23916070 10.1016/j.molimm.2013.06.013

[7] Huang C , Ying H , Yang X , et al. The clinical characteristics of other HLA‐B types in Chinese ankylosing spondylitis patients. Front Med. 2021;7:1‐10. 10.3389/fmed.2020.568790 PMC7820707 33490092

[8] Yang T , Duan Z , Wu S , et al. Association of HLA‐B27 genetic polymorphisms with ankylosing spondylitis susceptibility worldwide: a meta‐analysis. Modern Rheumatology. 2014;24(1):150‐161. 24261772 10.3109/14397595.2013.852856

[9] Díaz‐Peña R , Castro‐Santos P , Durán J , Santiago C , Lucia A . The genetics of spondyloarthritis. J Pers Med. 2020;10(4):E151. 33023259 10.3390/jpm10040151 PMC7711559

[10] Gonzalez‐Roces S , Alvarez MV , Gonzalez S , et al. HLA‐B27 polymorphism and worldwide susceptibility to ankylosing spondylitis. Tissue Antigens. 2010;49(2):116‐123. 10.1111/j.1399-0039.1997.tb02724.x 9062966

[11] Gonzalez S , Garcia‐Fernandez S , Martinez‐Borra J , et al. High variability of HLA‐B27 alleles in ankylosing spondylitis and related spondyloarthropathies in the population of Northern Spain. Hum Immunol. 2002;63(8):673‐676. 12121675 10.1016/s0198-8859(02)00404-4

[12] Dhaliwal JS , Too CL , Lisut M , Lee YY , Murad S . HLA‐B27 polymorphism in the Malay. Tissue Antigens. 2010;62(4):330‐332. 10.1034/j.1399-0039.2003.00107.x 12974801

[13] Ma HJ , Hu FP . Diversity of human leukocyte antigen‐B27 alleles in Han population of Hunan province, southern China. Tissue Antigens. 2010;68(2):163‐166. 10.1111/j.1399-0039.2006.00638.x 16866886

[14] Garcia‐Fernandez S , Gonzalez S , Mina Blanco , et al. New insights regarding HLA‐B27 diversity in the Asian population. Tissue Antigens. 2010;58(4):259‐262. 10.1034/j.1399-0039.2001.580407.x 11782278

[15] Bakland G , Gran JT , Nossent JC . Increased mortality in ankylosing spondylitis is related to disease activity. Ann Rheum Dis. 2011;70(11):1921‐1925. 21784726 10.1136/ard.2011.151191

[16] Voorter CE , Vlies S , Bergloonen E . Sequence‐based typing of HLA‐B: the B7 cross‐reacting group. Tissue Antigens. 2010;56(4):356‐362. 10.1034/j.1399-0039.2000.560408.x 11098936

[17] Zhang Z , Dai D , Yu K , et al. Association of HLA‐B27 and ERAP1 with ankylosing spondylitis susceptibility in Beijing Han Chinese. Tissue Antigens. 2014;83(5):324‐329. 24666027 10.1111/tan.12334

[18] Wang H , Zheng SP , Dang LJ , et al. Ankylosing spondylitis and HLA‐infections among b27‐carriers subtype correlation studies. Int J Med Lab. 2016;37(14):1954‐1958.

[19] Huang X , Mao W , Wang F , et al. Distribution of HLA‐B27 subtypes in Han population in Chongqing hematopoietic stem cell database. Chin J Blood Transfusion. 2006;19(4):321‐322.

[20] Zou HY , Yu WZ , Wang Z , He J , Jiao M . Human leukocyte antigen‐B27 alleles in Xinjiang Uygur patients with ankylosing spondylitis. Genet Mol Res. 2015;14(2):5652‐5657. 26125763 10.4238/2015.May.25.17

[21] Liu X , Hu LH , Li YR , Chen FH , Ning Y , Yao QF . The association of HLA‐B*27 subtypes with ankylosing spondylitis in Wuhan population of China. Rheumatol Int. 2010;30(5):587‐590. 19536542 10.1007/s00296-009-1018-0

[22] Yu XY , Gu GH , Gao C , et al. HLA‐B27 subtype is associated with ankylosing spondylitis. J Soochow Univ. 2006;4:605‐607.

[23] Liu Y , Jiang L , Cai Q , et al. Predominant association of HLA‐B*2704 with ankylosing spondylitis in Chinese Han patients. Tissue Antigens. 2010;75(1):61‐64. 19804562 10.1111/j.1399-0039.2009.01379.x

[24] Mou Y , Wu Z , Gu J , et al. HLA‐B27 polymorphism in patients with juvenile and adult‐onset ankylosing spondylitis in Southern China. Tissue Antigens. 2010;75(1):56‐60. 20196819 10.1111/j.1399-0039.2009.01406.x

[25] Fan CM , Huang RF , Chen GW . The association of HLA‐B27 genotype and subtype with ankylosing sponkylitis in Southern Fujian. Chin J Microeco. 2016;03:256‐258.

[26] Chou CT , Chen JM , Hsu CM , Chen SJ . HLA‐B27 and its subtypes in 4 Taiwanese Aborigine tribes: a comparison to Han Chinese patients with ankylosing spondylitis. J Rheumatol. 2003;30(2):321‐325. 12563689

[27] Hou TY , Chen HC , Chen CH , et al. Usefulness of human leucocyte antigen‐B27 subtypes in predicting ankylosing spondylitis: Taiwan experience. Intern Med J. 2010;37(11):749‐752. 10.1111/j.1445-5994.2007.01450.x 17908086

[28] Xu WH , Fu SM , Zhao Y . Study on the correlation between HLA‐B27 and its subtypes and ankylosing spondylitis in hainan area. Chin J Eugen Genet. 2008;06:29‐30.

[29] Huang JX , Wu Y , Huang Y , et al. Distribution and clinical significance of HLA‐B27 subtypes in ankylosing spondylitis patients of Han nationality in Fuzhou Area. Chin Foreign Med Res. 2018;16(34):9‐11.

[30] Danve A , Deodhar A . Axial spondyloarthritis in the USA: diagnostic challenges and missed opportunities. Clin Rheumatol. 2019;38(3):625‐634. 30588555 10.1007/s10067-018-4397-3

[31] Sesma L , Montserrat V , Lamas JR , Marina A , Vázquez J , López de Castro JA . The peptide repertoires of HLA‐B27 subtypes differentially associated to spondyloarthropathy (B*2704 and B*2706) differ by specific changes at three anchor positions. J Biol Chem. 2002;277(19):16744‐16749. 11875071 10.1074/jbc.M200371200

[32] Marsh S , Bodmer JG , Albert ED , et al. Nomenclature for factors of the HLA system, 2000. Tissue Antigens. 2010;57(3):236‐283. 10.1034/j.1399-0039.2001.057003236.x 11285132

[33] Gaalen F . Does HLA‐B*2706 protect against ankylosing spondylitis? A meta‐analysis. Int J Rheum Dis. 2012;15(1):8‐12. 22324941 10.1111/j.1756-185X.2011.01676.x

[34] Khan AM . Update: the twenty subtypes of HLA‐B27. Curr Opin Rheumatol. 2000;12(4):235‐238. 10910173 10.1097/00002281-200007000-00001

[35] Martin E , Sanz B , Guasp P . Separate effects of the ankylosing spondylitis associated ERAP1 and ERAP2 aminopeptidases determine the influence of their combined phenotype on the HLA‐B*27 peptidome. Autoimmun. 2017;79:28‐38. 10.1016/j.jaut.2016.12.008 28063628

[36] Robinson PC , Brown MA . Genetics of ankylosing spondylitis. Revista Médica De Chile. 2014;57(1):2‐11. 10.1016/j.molimm.2013.06.013 23916070

[37] Voruganti A , Bowness P . New Developments in our Understanding of Ankylosing Spondylitis Pathogenesis. Immunology. 2020;161(2):94‐102. 32696457 10.1111/imm.13242 PMC7496782

